# Integrated Causal Learning Workflow of Brain Iron Deposition in Patients with PD based on QSM

**DOI:** 10.2174/0115734056473262260817043352

**Published:** 2026-09-11

**Authors:** Wei Wei, Wuyan Zhao, Ying Yan, Zheng Wang, Hanzhu Jia, Yunlin Lei, Pengzhao Quan, Jiayun Wang, Mingjun Shi, Xu Yang, Shengjun Sun, Zhenchang Wang, Xuan Wei

**Affiliations:** 1 Medical Digital Intelligence Innovation Center, Beijing Friendship Hospital, Capital Medical University, Beijing, No. 95, Yong An Road, Xicheng District, Beijing 100050, China; 2 Department of Radiology, Beijing Friendship Hospital, Capital Medical University, Beijing, No. 95, Yong An Road, Xicheng District, Beijing 100050, China; 3 School of Computer Science, Beijing Institute of Technology, Beijing, No. 5, Zhongguancun South Street, Haidian District, Beijing 100081, China; 4 Department of Radiology, Beijing Tiantan Hospital, Capital Medical University, No. 119, South Fourth Ring West Road, Fengtai District, Beijing 100070, China; 5 Tangshan Research Institute, Beijing Institute of Technology, Beijing, No. 5, Zhongguancun South Street, Haidian District, Beijing100081, China; 6 Department of Urology, Beijing Friendship Hospital, Capital Medical University, Beijing, No. 95, Yong An Road, Xicheng District, Beijing 100050, China

**Keywords:** Parkinson's disease, Quantitative susceptibility mapping, Integrated causal learning workflow, Iron deposition, BAI

## Abstract

**Introduction:**

To investigate the model-inferred dependency pattern of iron deposition in the brain of patients with Parkinson's Disease (PD).

**Materials and Methods:**

Clinical and imaging data of 25 patients with PD and 36 Healthy Controls (HCs) were collected between January and December 2021. Iron deposition in the brain was quantified using Quantitative Susceptibility Mapping (QSM). An integrated causal learning workflow, combining GES-based causal discovery, causal effect estimation, and refutation analysis, was used to explore the causal relationships between clinical data and image characteristics and estimate their causal effects.

**Results:**

Beck Anxiety Inventory (BAI) and Beck Depression Inventory (BDI) scores were significantly greater in the PD group than the HC group (*P* = 0.003; *P* < 0.001). Timed Up and Go Test (TUG) and Berg Balance Scale (BBS) scores were lower in the PD group than in the HC group (*P* < 0.001; *P* < 0.001). The integrated causal learning workflow suggested a cross-sectional, model-inferred directional dependency structure with TH occupying an upstream position and downstream dependencies involving PUT, CN, RN, SN, and GP.

**Discussion:**

The iron deposition tissues in different regions of the brains of patients with PD exhibit a structured dependency pattern, which is of great significance for proposing hypotheses regarding the mechanism of iron deposition related to PD.

**Conclusion:**

The integrated causal learning workflow combined with QSM may help explore cross-sectional dependency patterns of brain iron deposition in patients with PD. These findings provide a biologically interpretable and hypothesis-generating workflow for understanding PD-related iron deposition patterns and may assist in early clinical detection of PD, rapid determination of its course, and timely administration of interventions and treatments.

## 1 INTRODUCTION

Parkinson's Disease (PD) is a common neurodegenerative disease in older people, which is characterized by resting tremor, bradykinesia, rigidity, and postural instability [1]. Iron is a redox-active metal required by the brain for a variety of physiological activities. Although the pathological hallmark of PD is believed to be abnormal folding and accumulation of α-synuclein in Lewy bodies in the brain [2], the disruption of iron homeostasis is known to lead to neurodegenerative diseases [3]. Previous studies have demonstrated that excessive deposition of free iron in brain cells is one of the key manifestations of PD, and the amount of iron deposition is correlated with the course of PD [4, 5].

Quantitative Susceptibility Mapping (QSM) can be used to quantitatively assess the content of paramagnetic substances such as iron and antimagnetic substances such as myelin, highlighting the structural contours and internal details of iron-containing tissues [6]. An increasing number of studies have applied QSM to determine brain iron content and have found that patients with PD show increased brain iron deposition, especially in the basal ganglia region [7, 8]. Patients at different stages of PD demonstrated varied patterns of iron deposition in corresponding brain regions [9]. Brain iron deposition in patients with PD is also associated with motor symptoms [10].

Dopamine analogues are commonly used clinically for the relief of PD symptoms. Depending on the response to the drug, patients are categorized into “ON” and “OFF” states. The “OFF” state is defined as 12 hours after the last PD drug treatment, and the “ON” state is defined as the use of super-ON doses of levodopa (125% of the morning dose) on the same day [11]. Patients with PD experience some control of their motor symptoms when they are in the “ON” state. To assess the degree of dyskinesia and its responsiveness to polycyclic amines, the present study evaluated exercise-related scale scores in patients with PD during the “ON” and “OFF” states. To minimize the impact of involuntary patient movement, the image acquisition was performed during the “ON” state.

Causal learning research can be broadly categorized into two main parts: causal discovery and causal inference. Causal discovery involves directly acquiring causal knowledge from observational data, while causal inference involves estimating the effects resulting from changes in specific variables on a desired outcome [12]. Previous researchers have utilized Dynamic Causal Modelling (DCM) and Granger Causality Analysis (GCA) to analyze Magnetic Resonance (MR) images of patients with PD to assist in understanding and quantifying physiological processes and neural responses in these patients [13] and to reveal brain iron deposition and its relationship to disease progression [9]. In this study, integrated causal learning workflow refers to an integrated analytical framework. The workflow combines several established components, including GES-based causal discovery, causal effect estimation, and refutation analysis. GES was used to learn the dependency structure among clinical and imaging variables, whereas regression-based estimation was used as one implementation tool for estimating model-implied effects [14]. Compared to DCM, this method does not require complex biophysical models or assumptions about interactions between brain regions. Additionally, our approach can uncover potential causal relationships among multiple variables and construct more complex causal structures, especially in scenarios with a larger number of variables.

In this study, we detected brain iron deposition in patients with PD using QSM and applied the integrated causal learning workflow to explore model-inferred directional dependencies among QSM-derived measures from different ROIs. We hypothesized that QSM-derived iron deposition measures across different brain regions may show a structured dependency pattern in patients with PD. Identifying such a dependency structure may provide hypothesis-generating information for understanding regional involvement in PD-related brain iron deposition. The aim of this study was to explore the cross-sectional dependency structure of QSM-derived brain iron deposition and its relationship with clinical features.

## 2 METHODS

### 2.1 Participants

Patients with PD were enrolled from Beijing Friendship Hospital, China and the patients received optimal treatment with dopaminergic medications.

The inclusion criteria were as follows:

(1) Between 40 and 80 years old;

(2) Diagnosed with PD for the first time and satisfied the diagnostic criteria of the PD Movement Disorder Society (MDS);

(3) Stable motor symptoms before enrolment;

(4) Ability to walk independently for a distance of 5 meters or more;

(5) Agreed to comply with the research protocol and were able to sign the informed consent form.

The exclusion criteria were as follows:

(1) Atypical or secondary parkinsonism;

(2) Abnormal mental state;

(3) Movement disorders caused by other neurological disorders.

### 2.2 Ethical Statement

This study was conducted in accordance with the Declaration of Helsinki (as revised in 2013). The study was approved by the ethics committee of Beijing Friendship Hospital, Capital Medical University, China (Approval No. 2019-P2-283-02). All included subjects signed an informed consent form.

### 2.3 Clinical Assessments

The required sample sizes were calculated through PASS 15.0, and all parameters were taken from previous experiments [15, 16]. We enrolled a total of 33 patients with PD and 50 healthy controls. Eight patients and 14 HCs were excluded due to inability to adhere to the scan, poor image quality, or missing data on clinical scales. Finally, 25 patients with PD and 36 HCs were included in the statistics. Hoehn-Yahr (H-Y) staging was used to evaluate disease progression in patients with PD [17]. H-Y staging was categorized into 5 stages, with stage one being the least symptomatic and stage five being the most symptomatic. Patients with PD with an H-Y score less than 2.5 were considered to have early-stage disease, and those with an H-Y score greater than or equal to 2.5 were considered to have advanced-stage disease. Patients with gait impairment were diagnosed through medical history and the MDS Unified -Parkinson Disease Rating Scale (MDS-UPDRS) assessment (a score ≥ 1 on Items 2.12 or 2.13 of the MDS-UPDRS-II or a score ≥ 1 on Items 3.10 or 3.11 of the MDS-UPDRS-III) [18]. The following scales were used to assess disease condition and gait disorders in patients with PD: the MDS-UPDRS, the Berg Balance Scale (BBS), the New Freezing of Gait Questionnaire (nFOGQ), and the Timed Up and Go (TUG) test. The MDS-UPDRS was used to comprehensively assess and rate disease severity. The BBS was used to measure the body’s balance. The TUG test was used to evaluate gait and the risk of falling during the test. All clinical evaluations were performed separately in both the “OFF” and “ON” states. Enrolled subjects were screened for intelligence by the Mini-Mental State Examination (MMSE) scale. The modified Apathy Evaluation Scale (MAES) was used to assess the severity of apathy in patients. The Beck Anxiety Inventory (BAI) was used to measure the degree of anxiety, and the Beck Depression Inventory (BDI) was used to measure the degree of depressive symptoms. All scales were graded by the same experienced clinical neurologist.

### 2.4 MRI Data Acquisition and Preprocessing

We used a 3.0T MRI system (Prisma, Siemens, Erlangen, Germany) to collect the image data. The collected sequences include high-resolution T1 structural sequences, QSM sequences, and Fluid-Attenuated Inversion Recovery (FLAIR) sequences. The specific parameters are as follows: 3D T1-Weighted (T1W) Magnetization Prepared Rapid Gradient Echo (MPRAGE) sequence for anatomic structure [repetition time (TR) = 7.3 ms, echo time (TE) = 2.4 ms, inversion time (TI) = 900 ms, flip angle = 9°, and isotropic voxel size = 1 mm^3^] and a 3D Multiecho Gradient Echo (ME-GRE) sequence for QSM (TR = 50 ms, first TE = 6.8 ms, TE interval = 4.1 ms, number of echoes = 10, flip angle = 15°, and voxel size = 1.5 × 1 .5× 2 mm^3^). The FLAIR sequences were used to detect abnormalities in the brain (TR = 8500 ms, TE =87 ms, TI = 2440, flip angle = 150°, and voxel size = 0.4 × 0.4 × 6 mm^3^).

### 2.5 Quantitative Susceptibility Mapping Preprocessing and Quantitative Analysis

The QSM data were preprocessed using the same protocol as that used in our previous study [16].

### 2.6 Selection of Regions of Interest

We chose the bilateral Red Nucleus (RN), Substantia Nigra (SN), Thalamus (TH), Putamen (PUT), Caudate Nucleus (CN), and Globus Pallidus (GP) as the Regions of Interest (ROIs). The ROIs were manually outlined by two experienced radiologists using ITK-SNAP software, and their susceptibilities were automatically extracted by the software. Neither of the two physicians was aware of the patient's group classification or clinical condition. The consistency of the delineation results of the two physicians was tested, and the Intraclass Correlation Coefficient (ICC) was 0.9558. The selected ROIs are shown in Fig. (**1**).

### 2.7 Statistical Analysis

We analyzed the data using SPSS 20.0 software (SPSS, Inc, Chicago, IL). Categorical variables in the demographic data and clinical scale scores are expressed as percentages. Continuous variables that conformed to a normal distribution are expressed as the mean ± standard deviation, and continuous variables that did not conform to a normal distribution are expressed as the median and interquartile range. For intergroup comparisons between the PD and HC groups, we used the two-independent-samples t test (for normally distributed data) or the rank-sum test (for nonnormally distributed data).

### 2.8 Integrated Causal Learning Workflow

The overall structure of the integrated causal learning workflow is depicted in Fig. (**2**). In this study, our workflow was defined as an integrated analytical framework rather than a newly developed causal discovery algorithm. This workflow consisted of GES-based causal discovery and causal effect estimation. The first component employed a two-stage greedy search procedure based on GES to learn the graph structure among clinical and imaging variables. The second component used causal inference principles to identify and estimate model-implied effects. Specifically, using linear regression models, the causal effect values are fitted.

Preprocessing of the data begins with filling in missing values. Mean imputation is used for QSM values, while samples with excessive missing values are removed. Subsequently, the original data are normalized to ensure each feature value is scaled to 0-1, which prepares them for the model input.

The two-stage greedy search causal algorithm is based on the Greedy Equivalence Search (GES) [14]. The process begins with a forward search, where each clinical indicator is treated as a feature node. The algorithm starts with a graph with no edges and incrementally adds edges to increase the model’s complexity. In each step, all possible edge addition operations are attempted, and the change in criterion score after each addition is calculated. The operation that minimizes the score is selected to update the graph structure. This process continues until no further operation can reduce the score. The backwards search phase aims to reduce model complexity and prevent overfitting. This stage starts with the graph from the forwards search and removes edges that change the score until a new minimum score is reached. After these two steps, a graph representing model-inferred directional dependencies among the included variables was generated under the assumptions of structural causal modeling.

In this study, the two-stage greedy search causal algorithm was applied to a graph with no edges, using various clinical indicators, including participants’ age, clinical scale scores, and the QSM values of the left and right CN, PUT, TH, GP, SN, and RN as nodes. Age was included as an observed candidate node in the structure-learning procedure, and in the subsequent effect-estimation step, age was also considered as a potential confounder when identifying adjustment sets for QSM-related effects. The Bayesian Information Criterion (BIC) score was used to assess the fit of the graph structure to the dataset [19]. The number of consecutive edges was repeatedly adjusted, and the graph with the lowest score was selected as the causal graph. Since some scales (*e.g*., gait data) were not causally related to the topic (QSM) studied in this paper, these scale items were removed from the causal graph, and the final model included participant age, BAI, BDI, and MMSE scores, and QSM values for each ROI.

Based on the causal graph, we selected treatment and outcome variables from the graph to estimate the causal effect. The treatment variable was actively changed, and the effect of this change on other variables was observed. The outcome variable was observed after the treatment variable was altered. We used clinical data, treatment variables, outcome variables, and causal graphs to construct a causal model in preparation for subsequent causal effect estimation. Subsequently, causal inference was performed based on the learned graph to identify and estimate model-implied effects between selected treatment and outcome variables. Regression-based estimation was used as one implementation tool for estimating the Average Treatment Effect (ATE). To evaluate the robustness of the learned graph structure, a resampling-based edge stability analysis was performed within our workflow. In each iteration, 95% of the original samples were randomly selected, and the GES procedure was repeated with the same model settings. This procedure was repeated 20 times, and the recovery frequency of each edge was calculated. Edges with a recovery frequency ≥ 0.50 were considered to have acceptable resampling support and were retained as relatively stable edges.

### 2.9 Refutation Tests

We conducted refutation tests to verify the correctness and robustness of our hypotheses. We employed two types of refutation tests: the add random common cause test and the placebo treatment test [20]. For the first test, if the hypothesis is correct, the causal effect should not change significantly after adding independent random variables as confounding factors to the dataset. For the second test, if the hypothesis is correct, the causal effect should be close to 0 after replacing the treatment variable with an independent random variable.

## 3 RESULTS

### 3.1 Demographics and Behaviors Measures of the Participants

The clinical information of the two groups is shown in Table **1**. Patients in both groups were comparable regarding sex. The PD group was older than the HC group (*P* = 0.007). BAI and BDI scores were significantly greater in the PD group than in the HC group (*P* = 0.003; *P* < 0.001). The TUG and BBS scores were significantly lower in the PD group than in the HC group (*P* < 0.001; *P* < 0.001). In the PD group, the MDS-UPDRS-III scores, MDS-UPDRS total scores, and TUG scores were significantly lower during the “ON” stage than during the “OFF” stage (*P =* 0.001; *P* < 0.001; *P* < 0.001), whereas the BBS scores were significantly higher than those of the “OFF” stage (*P* < 0.001).

### 3.2 Imaging of Patients with Early- and Late-stage PD

We selected two patients from the PD group, one at the mildest and one at the most severe H-Y stage, who were of similar age. Comparison of the QSM images of these two patients revealed increased iron deposition in the PUT, CN, TH, SN, and RN in the early-stage patient, while the iron content of individual grey matter nuclei was significantly greater in the late-stage patient, especially in the SN and GP. The image results were consistent with our findings (Fig. **3**).

### 3.3 The Result of Causal Graph

The graph derived from our workflow suggested a cross-sectional, model-inferred directional dependency structure among QSM-derived iron deposition measures. Within this structure, TH occupied an upstream position, with downstream dependencies involving PUT, CN/RN, SN, and GP. In addition to the QSM-related dependency structure, model-inferred directional dependencies were also observed among clinical variables, including BAI, BDI, and MMSE. The ATE values are shown in Fig. (**4**).

### 3.4 Causal Graph Refutation Tests

The results of refutation tests are shown in Table **2**. The causal effect does not change significantly after using the add random common cause test, and it is close to 0 after using the placebo treatment test. Therefore, the results in the table demonstrate the validity of our hypothesis.

### 3.5 Resampling-based Edge Stability Analysis

The stability analysis showed that multiple edges were consistently recovered across subsampling iterations. In particular, several ROI-to-ROI edges showed high stability, including CN_L → CN_R, CN_L → GP_R, GP_R → GP_L, SN_R → SN_L, PUT_R → RN_R, RN_R → SN_R, SN_R → GP_R, PUT_L → PUT_R, PUT_R → CN_L, TH_L → PUT_L, and TH_L → RN_R. These findings indicate that the main QSM-derived dependency structure was generally preserved under repeated subsampling. The results are displayed in Table **3**.

## 4 DISCUSSION

In this study, we explored the cross-sectional, model-inferred dependency pattern of QSM-derived brain iron deposition in patients with PD using an integrated causal learning workflow. The causal graph suggested that TH and PUT occupied upstream positions in the dependency structure, with downstream dependencies involving CN/RN, SN, and GP. Our findings were consistent with those reported by Su Yan *et al*. on brain iron deposition using GCA [9]. A review has summarized that the extent of iron deposition is related to the progression of PD [21]. Increased brain iron deposition in PD individuals led to the loss and excessive depletion of dopaminergic neurons, disrupting neural circuits in the basal ganglia and leading to motor and non-motor symptoms, including mood disorders and cognitive deficits [22].

Our research team had analyzed the same data in a previous study, in which we compared changes in brain iron deposition between patients with PD and normal subjects and examined differences in brain iron content between patients with and without Freezing of Gait (FOG) [16]. In addition, we correlated brain iron deposition in patients with PD with clinical scale scores and objective gait data. We found that patients with PD have greater brain iron deposition than normal subjects do. Increased iron deposition in the SN was associated with motor deficits in FOG patients [16]. However, due to the lack of specificity of symptoms in the early stages of PD, it is difficult to detect and treat the disease in a timely manner. The organization of iron deposition across different brain regions in patients with PD may show structured dependency patterns. Therefore, developing an analytical approach to explore such regional dependency structures may be valuable for generating hypotheses about PD-related iron deposition involvement. Previous reports have primarily used DCM to study the brains of patients with PD [13, 23]. Their model primarily analysed causal relationships in time-series data, inferring dynamic changes in brain connectivity during specific tasks or stimuli by modelling effective connections in resting-state magnetic resonance imaging (rs-fMRI) data. Such a method provides observations on the dynamics of brain networks, focusing on understanding and quantifying physiological processes and neural responses. The GCA was utilized to explore the neural mechanisms underlying apathy in patients with PD [24], and studies have also employed GCA on pseudo-time series MRI data to reveal the relationship between brain iron deposition in patients with PD and disease progression [9]. However, both methods require complex biophysical modelling and assumptions about interactions among brain regions, making the analysis of the sequence of brain iron deposition in PD cumbersome, complex, and inefficient. In view of this, we applied the integrated causal learning workflow to explore model-inferred directional dependencies among brain iron deposition measures. This workflow provides a structured and hypothesis-generating approach, avoids the need to construct complex biophysical models or impose assumptions about interregional interactions, and substantially simplifies the assessment procedure while improving analytical efficiency.

### 4.1 Brain Iron in the TH as an Upstream Node in the Learned Graph

One of the most striking differences in PD is the change in thalamic neuronal activity in motor circuits. Some of these changes resulted indirectly from pathology in the basal ganglia region, and others were caused by pathology in the TH itself [25]. The TH is not only a key relay node of the thalamocortical circuit but also a center for gait regulation. The dynamic functional connectivity between the thalamus and the cortex was disrupted in PD patients with FOG [26]. Changes in the structural and functional connectivity of the TH can be used to characterize different motor subtypes of PD, and distinct subregions can serve as targets for electrical stimulation in PD treatment [27-29]. In addition to motor function, iron deposition in the TH was also correlated with cognitive decline in patients with PD [30]. Although numerous studies have demonstrated that PD patients exhibit structural and functional damage and increased iron deposition in the TH, no report has focused on the correlation between iron deposition in the TH and the timeline of disease progression. Our research has revealed that the iron deposition in the TH occurs upstream of the causal pathway. This finding may be partially because the TH belongs to the input nucleus of the basal ganglia. The thalamic synapse is one of the striatal afferent pathways, although it has often been overlooked in various studies [31]. This conclusion needs to be validated by further research.

### 4.2 The Iron Deposits in PUT, SN and CN Occur Downstream of TH in the Learned Graph

No clear causal relationship was found between SN and CN. One study revealed that the morphology of the PUT in patients with early-onset PD differed from that of HCs and was more pronounced on the right side of the PUT [32]. This finding suggests that the PUT undergoes structural changes early in PD. In addition, low PUT volume may be an important clinical indicator of motor dysfunction and neurological symptoms in patients with PD [33]. One study revealed that half of patients with PD presented early with significant dopaminergic dysfunction in the CN [34]. Iron deposition in the striatal system was directly associated with abnormal dopaminergic function in patients with PD [35]. Iron deposition in the RN has been shown to occur in patients with early PD, and patients with H-Y stage II disease had significantly more iron in the RN than controls [36]. Another study suggested that the course of PD was associated with increased RN volume [37]. However, it has also been shown that altered iron levels in the RN are involved in the late stage of PD [38]. In fact, the increased iron levels in the RN are a dynamic and cumulative process in which every disease process is involved. Early abnormalities in dopamine function within the SN nuclei cause a range of symptoms. PD patients with gait disturbances had significantly greater susceptibility to iron deposition in the PUT, RN, and CN than PD patients without gait disturbances [39]. Loss of dopaminergic neurons projecting to the caudate nucleus was correlated with early deficits in memory, mild cognitive impairment, and musculoskeletal pain in patients with PD [40, 41].

### 4.3 The Iron Deposits in the SN And GP Occur at the Downstream Stage of the Causal Model

Increased iron levels in the SN were a powerful biomarker for advanced PD [42, 43]. In our study, the iron deposition in PD occurs at the downstream stage of the causal model, indicating that it is influenced by multiple upstream factors. Iron deposition in the substantia nigra pars compacta (SNc) occurs in the early stages of PD, while iron deposition in the substantia nigra pars reticulata (SNr) occurs in the late stages of PD. Since the SNc is the main damaged structure in PD patients, this region may be more susceptible to iron accumulation than other brain regions [44]. Similarly, other studies have shown that iron deposition in the SNc was observed only in patients with early PD, whereas iron deposition in the SNr was observed only in patients with advanced PD [45]. Our study did not conduct an internal division of the structure of the SN, which is one of the limitations of the research. However, a recent study has shown that although iron deposition in the SN of PD patients increases slowly with the course of the disease until it reaches a stable level in the late stages, this increase in iron content may be closely related to the use of PD medications [46]. This is a significant departure from previous studies. Therefore, whether the increase in iron content in SN is a pathological change in the disease itself or an effect of the drugs needs to be further explored by more studies.

The GP, especially the internal segment (GPi), exhibits iron deposition in the advanced stages of the disease [44]. In patients with advanced PD, elevated iron levels spread more widely into the GP and RN than in those with early PD. Increased iron levels in these regions impair behavioral functions during normal ageing [47]. A study showed a strong correlation between QSM values and disease stage in GP [48]. Since the majority of patients in this study were at an advanced stage of the disease, the iron deposition in the GP, which is located at the very bottom of the causal model, may also be related to disease stage.

### 4.4 Causal Relationship in the Iron Deposition Between the Left and Right Sides Within Each ROI

There is no causal relationship between the iron deposits on the left and right sides, indicating that the iron deposits on both sides are separate processes and do not influence each other. Similarly, Sun *et al*. reported increased iron in the SN bilaterally in patients with isolated REM Sleep Behavior Disorder (iRBD) and PD [49]. In line with that, He *et al*. reported increased iron in the SN bilaterally in patients with stage I disease [50]. Our results showed that iron deposition in the SN in patients with iRBD and PD is not a significant problem. Thus, although patients showed Parkinsonian symptoms only on one side of the body at the earliest clinically detectable stage, disrupted iron deposition was also observed in the SN on the asymptomatic side [36].

## 5 STUDY LIMITATIONS

There are some limitations in the study. First, the age of our included PD patients differed significantly from that of the HCs, which may affect the statistical results for patient demographics but did not affect the results of the causal analyses. Second, we selected only classical grey matter nuclei as ROIs for data extraction and analyses; herein, we need to perform further voxel-level data analysis. Finally, this was a cross-sectional study in which data from patients with PD at different stages were collected during the same period. Further information on longitudinal changes in each patient needs to be collected for own-control studies if necessary. Despite these limitations, we presented an integrated causal learning workflow, in conjunction with QSM sequences, to explore directional dependency structures among QSM-based regional susceptibility measures in PD and for estimate model-implied effects.

## CONCLUSION

In summary, QSM can be used as a reliable imaging tool to assess cerebral iron deposition in patients with PD. The integrated causal learning workflow combined with QSM may help explore cross-sectional dependency patterns of brain iron deposition in patients with PD. These findings provide a biologically interpretable and hypothesis-generating workflow for understanding PD-related iron deposition patterns. These indicators help assess the condition of PD patients, especially in early-stage patients, and guide the clinic in taking timely treatment and intervention measures.

## Figures and Tables

**Fig. (1):**
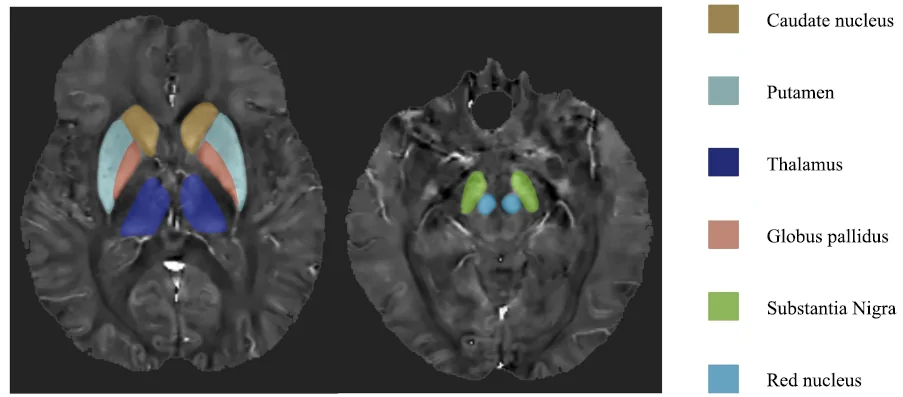
The bilateral red nucleus, substantia nigra, thalamus, putamen, caudate nucleus, and globus pallidus were chosen as ROIs. Adapted from Wei *et al*. under the terms of the Creative Commons CC BY 4.0 license [16].

**Fig. (2):**
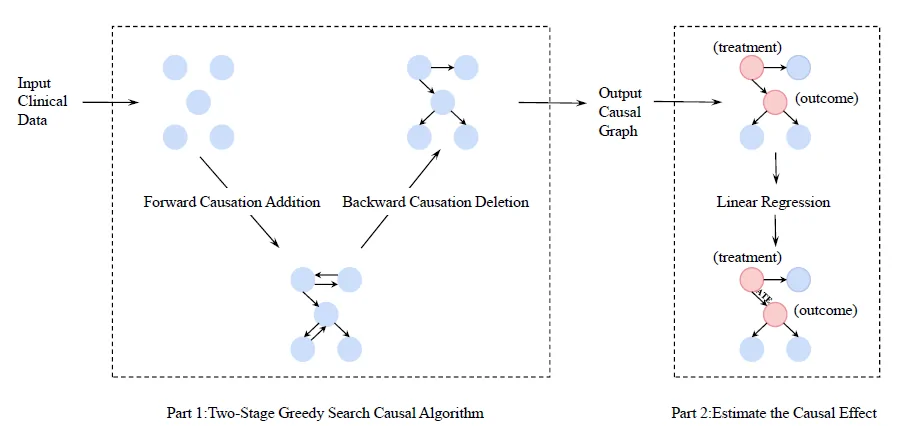
The illustration graph for our workflow. Taking clinical data as the input of the workflow, the first part of the workflow employs a two-stage greedy search causal algorithm to perform forward causation addition and backward causation deletion on clinical data, resulting in the optimal causal graph fitted to the clinical data. Subsequently, the second part of the workflow is performed on the causal graph; that is, the treatment and outcome variables in the causal graph are selected, and linear regression is used to estimate the causal effect of the treatment variable on the outcome variable to obtain the ATE value.

**Fig. (3):**
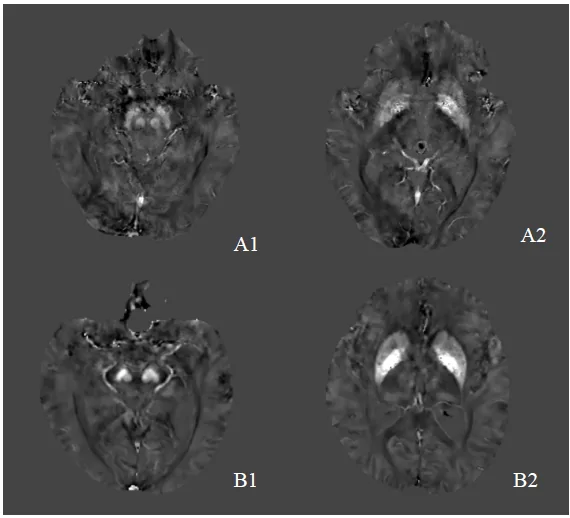
QSM images of two patients with PD. A1 and A2 are patients with H-Y stage 1. B1 and B2 are patients with H-Y stage 4. As can be seen from the figure, patients in stage 4 had higher susceptibilities in all basal ganglia regions than patients in stage 1. The bilateral substantia nigra and globus pallidus are particularly well represented.

**Fig. (4):**
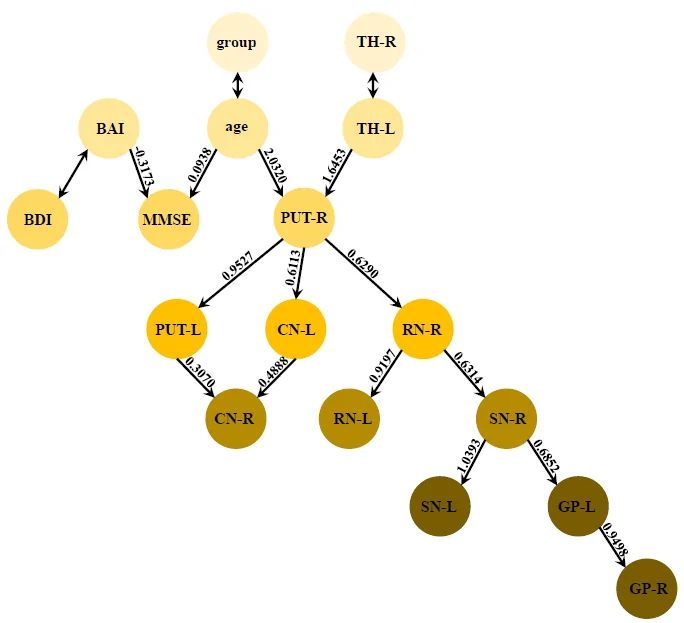
Diagram of the results of the integrated causal learning workflow. One-way arrows indicate unidirectional causation and two-way arrows indicate bidirectional causation. Numerical values are average treatment effect, with larger values indicating a stronger causal relationship between variables. The initial site of cerebral iron deposition in PD patients was the TH, followed by PUT, CN, and RN. There was no significant difference in the deposition of CN and RN across the different disease stages. The SN and the GP, on the other hand, appear in the later stages of PD. Anxiety and depression are causal to each other. The higher the anxiety value, the lower the cognitive performance.

**Table 1:** Demographic and clinical characteristics of the HC and PD participants.

Measurements	HC	PD	
		PD Clinical parameters	
		ON State	OFF State
Total	36	25	
Age (year)	64.5(55.5, 70.0)	68.0 (64.0, 72.5)**	
Sex (% male)	15(41.7%)	7 (28.0%)	
Education (> 9year)	16(44.4%)	13 (52.0%)	
Duration of disease (months)	n/a	57.0±32.9	
LEDD (mg)	n/a	610.9±298.2	
H-Y (>2.5)	n/a	6 (24.0%)	
MMSE	28.0 (25.3, 29.0)	28.0 (28.0, 29.0)	
BAI	24.0 (22.0, 26.0)	26.0 (24.5, 30.0)**	
BDI	3.0 (1.0, 4.8)	9.0 (4.0, 10.0)**	
NFOGQ	n/a	11.0 (44.0%)	
PDQ-39	n/a	26.3±18.4	
MDS-UPDRS-III	n/a	18.4±8.5	30.5±12.2^##^
MDS-UPDRS Total	n/a	42.0±19.0	54.1±22.7^##^
TUG	8.2 (7.3, 8.8)	10.2 (8.5, 12.2)**	11.7 (9.8, 15.3)**^##^
BBS	56.0 (54.0, 56.0)	52.0 (45.0, 54.0)**	49.0 (36.5, 52.5)**^##^

**Abbreviations:** HC, heathy control; PD, Parkinson’s disease; MMSE, Mini-Mental State Examination; BAI, Back Anxiety Inventory; BDI, Back Depression Inventory; LEDD, levodopa equivalent daily dose (mg); H-Y, Hoehn-Yahr score; NFOGQ, New Freezing of Gait Questionnaire; PDQ-39, Quality of life questionnaire for patients with Parkinson's Disease; MDS-UPDRS, International Parkinson and Movement Disorder Society-Unified Parkinson’s Disease Rating Scale; MDS-UPDRS-III, MDS-UPDRS motor score; TUG, Timed up and Go Test; BBS, Berg Balance Scale. Same set of clinical data from the control was used to analyze against PD group.

**P < 0.01, compared with control group.

^##^ *P* < 0.01, compared with ON State.

**Table 2:** Causal effect values and refutation test values.

Causal Direction	ATE	Refutation Test- add random common cause	*P*-value	Refutation Test- placebo treatment	*P*-value
BAI->MMSE	-0.3173	-0.3176	0.98	0.0071	0.98
age->MMSE	0.0938	0.0933	0.98	0.0010	1.0
age->PUT-R	2.0320	2.0368	0.96	0.0131	0.98
th-L->PUT-R	1.6453	1.6439	0.96	0.0139	0.96
PUT-R->PUT-L	0.9527	0.9527	0.98	0.0065	0.96
PUT-R->CN-L	0.6113	0.6117	0.96	0.0042	0.96
PUT-R->RN-R	0.6290	0.6294	0.96	0.0114	0.96
PUT-L->CN-R	0.3070	0.3067	0.96	0.0036	0.98
CN-L->CN-R	0.4888	0.4879	0.96	0.0083	0.98
RN-R->RN-L	0.9197	0.9201	0.96	0.0117	0.98
RN-R->SN-R	0.6314	0.6320	0.96	0.0015	0.96
SN-R->SN-L	1.0393	1.0393	0.98	0.0011	0.98
SN-R->GP-L	0.6852	0.6851	0.96	0.0042	0.98
GP-L->GP-R	0.9498	0.9503	0.96	0.0071	1.0

**Abbreviations:** ATE, average treatment effect; MMSE, Mini-Mental State Examination; BAI, Back Anxiety Inventory; RN, red nuclei; SN, substantia nigra; TH, thalamus; GP, globus pallidus; CN, caudate nucleus; PUT, putamen.

**Table 3:** Edges with recovery frequency ≥ 0.50 in the resampling-based stability analysis.

Recovery frequency	Count / 20	Stable edges
1.00	20 / 20	CN_L → CN_R; CN_L → GP_R; GP_R → GP_L; SN_R → SN_L; TH_L → gender
0.95	19 / 20	BAI → gender; PUT_R → RN_R; RN_R → SN_R; SN_R → GP_R
0.90	18 / 20	BDI → BAI; PUT_L → PUT_R; PUT_R → CN_L; RN_R → RN_L; age → PUT_L; group → BDI; TH_L → PUT_L; TH_L → RN_R
0.85	17 / 20	PUT_L → CN_R
0.80	16 / 20	GP_L → group; SN_R → group
0.75	15 / 20	MMSE → BDI
0.70	14 / 20	TH_R → RN_L
0.65	13 / 20	MMSE — age
0.50	10 / 20	age → TH_R; TH_L → TH_R

**Abbreviations:** CN, caudate nucleus; GP, globus pallidus; SN, substantia nigra; TH, thalamus; PUT, putamen; RN, red nucleus; L, left; R, right. “→” indicates a directed edge, whereas “—” indicates an undirected edge. Edges with a recovery frequency ≥ 0.50 were considered to have acceptable resampling support. The variable “gender” refers to the coded sex/gender variable used in the model.

## Data Availability

The corresponding authors [X.Y, S.S, Z.W, X.W] graciously offer accessibility to the data utilized in this study, ensuring their availability upon the submission of reasonable inquiries. It is important to note that, in order to safeguard privacy, precautions may be implemented to regulate access to sensitive data. The underlying code for this study [and training/validation datasets] is not publicly available for proprietary reasons.

## LIST OF ABBREVIATIONS

PD: Parkinson's Disease
QSM: Quantitative Susceptibility Mapping
HC: Healthy Control
BAI: Beck Anxiety Inventory
BDI: Beck Depression Inventory
TUG: Timed Up and Go Test
BBS: Berg Balance Scale
MR: Magnetic Resonance
MDS-UPDRS: MDS Unified Parkinson's Disease Rating Scale
nFOGQ: New Freezing of Gait Questionnaire
MMSE: Mini-Mental State Examination
MAES: Modified Apathy Evaluation Scale
RN: Red Nucleus
SN: Substantia Nigra
TH: Thalamus
PUT: Putamen
CN: Caudate Nucleus
GP: Globus Pallidus
ROI: Region of Interest
ICC: Intraclass Correlation Coefficient
GES: Greedy Equivalence Search
BIC: Bayesian Information Criterion
ATE: Average Treatment Effect
FOG: Freezing of Gait
iRBD: Isolated REM Sleep Behavior Disorder

## STANDARDS OF REPORTING

STROBE guidelines were followed.
